# Dose–response and recovery trajectories of fall prevention rehabilitation in older patients with vertigo: a retrospective cohort study based on vestibular and gait impairment phenotypes

**DOI:** 10.3389/fneur.2026.1874677

**Published:** 2026-08-17

**Authors:** Xiao-Qing Zhang, Jing Qin, Hong-Min Wang, Li Liu, Jie Zhang, Ya-Hui Wang, Wen-Ting Wang, Rui-Ting Zhu, Hong-Lei Guo

**Affiliations:** 1 Department of Neurology, The First Hospital of Hebei Medical University, Shijiazhuang, Hebei, China; 2 Department of Rehabilitation, The First Hospital of Hebei Medical University, Shijiazhuang, Hebei, China

**Keywords:** dose–response, fall prevention, neuro-otology, older adults, timed up and go test, trajectory analysis, vertigo, vestibular rehabilitation

## Abstract

**Background:**

Vertigo and dizziness are common in older adults and are associated with a significantly increased risk of falls. Fall prevention rehabilitation therapy (FPRT) is recommended, but current prescriptions remain largely empirical and do not account for heterogeneity in baseline vestibular and gait impairments. The dose–response relationship between rehabilitation hours and functional improvement, and how baseline phenotypes are associated with recovery trajectories, are poorly understood.

**Methods:**

This retrospective longitudinal cohort study, conducted in accordance with STROBE guidelines, included 756 older patients with vertigo (mean age 68.4 ± 8.2 years) who completed a standardized 10-week FPRT program. The primary outcome was the change in Timed Up and Go (TUG) test over 10 weeks. Group-based trajectory modeling (GBTM) was used to identify distinct functional recovery trajectories. Restricted cubic splines (RCS) within generalized estimating equation models were used in a planned exploratory secondary analysis to explore nonlinear dose–response relationships between time-varying cumulative rehabilitation hours and achievement of the minimal clinically important difference (MCID).

**Results:**

Three distinct recovery trajectories were identified: rapid responders (34.1%), gradual responders (47.5%), and poor responders (18.4%). Baseline impairment phenotypes were associated with trajectory membership. Patients with isolated vestibular impairment were enriched among Rapid Responders, whereas age ≥75 years (OR = 3.45, 95% CI = 2.12–5.61) and complex mixed impairment involving vestibular, dynamic gait, and proprioceptive deficits (OR = 4.85, 95% CI = 2.95–7.98) were independently associated with Poor Responder membership. The dose–response relationship was nonlinear. The probability of achieving MCID (≥80%) required 35–40 cumulative hours for rapid responders, 55–60 h for gradual responders, and showed a plateau with minimal benefit beyond 70 h for poor responders.

**Conclusions:**

Baseline vestibular, dynamic gait, and proprioceptive impairment phenotypes were associated with longitudinal recovery trajectories and exploratory dose–response patterns in older patients with vertigo. The observed 80% MCID probability points, approximately 35–40 h for Rapid Responders and 55–60 h for Gradual Responders, should be interpreted as hypothesis-generating estimates rather than definitive prescription targets. These findings may inform future prospective studies of phenotype-informed fall prevention rehabilitation.

## 1 Introduction

Vertigo and dizziness are among the most frequent clinical presentations in neurological practice, with a high prevalence reported in the general population (1). These symptoms profoundly disrupt spatial orientation and postural control, leading to significant functional impairments. The most critical consequence is the dramatically elevated risk of falls. Epidemiological data consistently show that falls are a leading cause of severe trauma, morbidity, and mortality, especially among individuals aged 65 and older, with an annual incidence ranging between 10 and 25% in community-dwelling older adults (2). This generates immense healthcare burdens globally. Importantly, vestibular dysfunction specifically exacerbates this vulnerability. A recent systematic review and meta-analysis confirmed that older adults with dizziness have a significantly higher risk of future falls and fall-related injuries (3). Consequently, mitigating fall risk in patients with vertigo through systematic evaluation and targeted intervention has emerged as a critical global public health imperative (4).

In response to this pressing clinical challenge, Fall Prevention Rehabilitation Therapy (FPRT) has garnered substantial attention (5). In our institution, we utilize a locally adapted framework termed FPRT, which integrates classic Cawthorne–Cooksey exercises with contemporary, globally recognized vestibular rehabilitation therapy (VRT) guidelines (6). Unlike passive therapeutic modalities, FPRT employs an active, systemic coordination rehabilitation model that aims to harness the inherent plasticity of the central nervous system to foster vestibular compensation and enhance somatosensory integration (7, 8). Clinical guidelines, such as the World Guidelines for Falls Prevention and Management for Older Adults, increasingly advocate for early fall risk screening and the subsequent implementation of targeted interventions like FPRT (9). The efficacy of FPRT is particularly pronounced in patients with vestibular dysfunction and incomplete functional impairments, a demographic that constitutes the vast majority of vertigo cases in neurological settings (5, 10). By intervening when patients exhibit a potential risk of falling—prior to the occurrence of a catastrophic fall event—FPRT serves as a highly effective secondary prevention strategy (9). However, despite the acknowledged benefits of FPRT, a significant knowledge gap persists regarding its precision application, optimal protocols, and long-term outcomes (5, 11).

Currently, rehabilitation prescriptions for vertigo remain largely empirical and often employ a generic, “one-size-fits-all” approach (12). Conventional recommendations vaguely suggest a therapeutic course of at least 10 weeks, accumulating no less than 50 h of total training (12, 13). In real-world clinical settings, however, neurologists observe profound heterogeneity in patient responses to this standardized regimen (14, 15). A subset of patients exhibits rapid functional restoration within a few weeks, while others, despite rigorous adherence to the protocol, experience protracted recovery or minimal clinical benefit (15). We hypothesize that this observed heterogeneity is not random but is intrinsically linked to the patient's baseline “impairment phenotype”—the specific combination and severity of vestibular deficits, dynamic gait abnormalities, and concurrent sensorimotor declines (16, 17). For instance, a patient with isolated unilateral vestibular hypofunction may possess a fundamentally different functional recovery trajectory and require a different cumulative training dose compared to an elderly patient with concurrent dynamic visual acuity loss, proprioceptive deficits, and severe gait instability (18, 19).

Understanding the distinct recovery trajectories of patients with vertigo and exploring potential dose–response patterns may help inform more individualized vestibular rehabilitation strategies (20). Characterizing these potential dose–response patterns may also support the development of specialized fall prevention clinics and more efficient allocation of rehabilitation resources (6). Therefore, the primary objective of this retrospective longitudinal cohort study is to map the dynamic functional recovery trajectories of patients with vertigo undergoing customized vestibular rehabilitation. Utilizing comprehensive real-world data from 2024 to 2025, we aim to quantify how baseline vestibular and dynamic gait impairment patterns determine the threshold of rehabilitation dose (quantified primarily as cumulative active training hours) required to achieve a minimal clinically important difference (MCID) in patient-reported outcomes (21). By doing so, this study endeavors to transition vestibular rehabilitation from an empirical, one-size-fits-all practice toward a precision medicine paradigm that tailors intervention based on individual patient profiles and recovery pathways (22).

## 2 Methods

### 2.1 Study design and setting

This is a retrospective, longitudinal cohort study designed to evaluate the trajectories of functional recovery in patients with vertigo receiving FPRT. The study was conducted at the Department of Neurology, The First Hospital of Hebei Medical University, a tertiary academic medical center in Hebei, China. We extracted and analyzed clinical data from patients admitted between January 1, 2024, and December 31, 2025. The study protocol was approved by the Institutional Review Board and Ethics Committee of The First Hospital of Hebei Medical University. Due to the retrospective nature of the analysis, the requirement for direct informed consent for this specific data extraction was waived; however, all included patients had previously provided written informed consent for their clinical data to be utilized for research purposes at the time of admission. This study was reported in strict adherence to the Strengthening the Reporting of Observational Studies in Epidemiology (STROBE) statement guidelines to ensure methodological transparency and rigor (23).

### 2.2 Participants and eligibility criteria

The source population consisted of consecutive patients admitted to the neurology ward with a primary chief complaint of vertigo or dizziness. To ensure a homogeneous cohort capable of safely participating in and benefiting from FPRT, strict eligibility criteria were applied. Inclusion criteria were defined as follows: (1) Confirmed balance and gait dysfunction primarily originating from the vestibular system [differentiated via comprehensive neuro-otological testing, including video head impulse testing (vHIT) and caloric testing, distinguishing peripheral from specific central etiologies], presenting a demonstrable risk of falls (defined as TUG > 12s or FRQ score ≥ 4); (2) Activities of Daily Living (ADL) score ≥ 41 points, indicating sufficient baseline independence to engage in physical rehabilitation; (3) Ability to fully cooperate with the baseline FPRT assessment; (4) Clear consciousness with no severe cognitive impairment [screened via the Mini-Mental State Examination (MMSE)] (24); and (5) Completion of at least 10 weeks of follow-up with a minimum of three distinct functional assessment time points. Exclusion criteria included: (1) Concomitant severe cardiopulmonary diseases, advanced malignancies, or other critical illnesses precluding physical exertion; (2) ADL score ≤ 40 points; (3) Baseline assessments indicating absolute contraindications for FPRT; (4) Incomplete clinical data or loss to follow-up before the 10-week endpoint; (5) Severe communication barriers (e.g., dementia, psychiatric disorders, delirium); and (6) A documented history of active substance or medication abuse.

### 2.3 Baseline assessment and impairment phenotyping (exposure and covariates)

Upon admission, all patients underwent a comprehensive, multidisciplinary baseline fall risk and functional assessment. Demographic and clinical covariates extracted included age, sex, educational level, disease duration, lifestyle factors, and the Charlson Comorbidity Index (CCI) (25) to quantify the burden of comorbid conditions. Mental health status was evaluated using the Hospital Anxiety and Depression Scale (HADS) (26) and the Geriatric Depression Scale (GDS) (27). Cognitive function was screened via the Mini-Mental State Examination (MMSE) (24).

The core independent variables (exposures) pertained to the baseline impairment phenotype, systematically evaluated through a battery of validated instruments: (1) vestibular and balance dysfunction: quantified using the Dizziness Handicap Inventory (DHI) (28), Vestibular Disorders Activities of Daily Living Scale (VADL) (29), tandem stance test, and specialized vestibular and oculomotor testing (including static and dynamic visual acuity). (2) Dynamic gait and mobility: assessed primarily via the Timed Up and Go (TUG) test (30), Dynamic Gait Index (DGI) (31), and the Functional Reach (FR) test. (3) Muscle strength and coordination: evaluated using the five times sit to stand test (FTSST) (32) and single leg stance (SLS) time (33). (4) Proprioception and sensory integration: evaluated utilizing the modified Clinical Test of Sensory Interaction on Balance (mCTSIB) (34) and clinical assessment of joint position sense. (5) Fall risk confidence: measured using the activities-specific balance confidence (ABC) scale (35) and the fall risk questionnaire (FRQ) (36).

Based on these baseline metrics, we utilized a hierarchical clustering approach (detailed in the statistical analysis section) to categorize patients into distinct “Baseline Impairment Phenotypes,” differentiating between isolated vestibular deficits and complex, multi-systemic involvements (e.g., combined vestibular, proprioceptive, and dynamic gait impairments).

### 2.4 Fall prevention rehabilitation therapy (FPRT) protocol

The FPRT intervention employed in this study was an active, generalized coordination rehabilitation model, consistent with multimodal vestibular rehabilitation principles derived from global guidelines (37), tailored to individual baseline deficits. The core philosophy was progressive overload—starting from easy to difficult, and slow to fast, ensuring patient safety while gradually increasing intensity. Initial training commenced during hospitalization under the supervision of trained rehabilitation specialists, consisting of daily 45-min sessions for the first 5 to 7 days. During this phase, all patients were introduced to a standardized core set of exercises, which were subsequently modified in complexity and duration based on individual tolerance and phenotype. Upon discharge, patients were transitioned to a home-based program, aiming for approximately 5 h per week (roughly 45–60 min per session, 5 days per week). This phase was managed via a dedicated follow-up team utilizing WeChat groups for daily video check-ins and weekly remote reassessments. Exercise intensity was monitored using the modified Borg rating of perceived exertion (RPE), targeting a moderate intensity (RPE 3–4), with progression parameters adapted dynamically. Each supervised session consisted of a 5-min warm-up, 30–35 min of task-specific FPRT modules, and a 5-min cool-down. For each selected exercise, patients performed 2–3 sets of 8–12 repetitions or 30–60 seconds per set, with rest intervals adjusted according to symptoms and fatigue. Progression was permitted when patients completed two consecutive sessions at RPE ≤ 3 without near-falls, severe dizziness, or prolonged symptom exacerbation. Progression included narrowing the base of support, increasing head-turn speed, adding dual-task components, increasing walking complexity, or increasing exercise duration by approximately 10–15% per week. Exercise difficulty was reduced or temporarily suspended if RPE exceeded 5, if near-falls occurred, or if dizziness, nausea, or fatigue persisted for more than 24 h after training. Adequate adherence was defined *a priori* as completion of at least 80% of prescribed home sessions. Home-based adherence was calculated as the proportion of prescribed sessions verified by video check-ins and training logs. Patients with insufficient adherence or fewer than three follow-up assessments were excluded from the longitudinal trajectory analysis. The protocol mandated a minimum duration of 10 weeks and a target cumulative volume of no less than 50 h.

The specific FPRT modules included: (1) muscle strength rehabilitation**:** FTSST (enhancing quadriceps/ankle stability), SLS (strengthening weight-bearing capacity), and Heel-to-Toe Raise (HTR, targeting calf and intrinsic foot muscles). (2) Center of gravity (COG) shift rehabilitation: COG Shifts (CGS, rapid alternating leg raises), functional reach (FR, enhancing COG maintenance), and Gait with Pivot Turn (GPT, training response to sudden directional changes). (3) Balance and coordination: horse-stance with hand movements (LH), Lunge with ball passing (LB, integrating head-eye-limb coordination), and Heel-to-Toe Walking (HTW). (4) Dynamic gait rehabilitation: standard TUG training, tandem walking (TW), and the four-item dynamic gait protocol (walking under varying speeds and head-turn conditions) based on the DGI framework (31). The specific modules prescribed were highly individualized based on the baseline phenotype: phenotype A primarily focused on modules 1 and 3; phenotype B incorporated all modules with an emphasis on dynamic gait (module 4); and phenotype C required a cautious integration of all modules, specifically emphasizing proprioceptive and sensory reweighting exercises.

The “Dose” of FPRT was rigorously quantified as the cumulative active training time (in hours) recorded via the supervised inpatient logs and verified home-based video daily check-ins over the 10-week period.

### 2.5 Outcome measures

The primary outcome was the longitudinal trajectory of TUG performance from baseline to week 10. Secondary outcomes included longitudinal DHI change, the incidence of fall events during the 10-week follow-up, and achievement of the minimal clinically important difference (MCID). The TUG test was selected for its robust psychometric properties in evaluating dynamic balance and mobility, while the DHI provided a subjective measure of vestibular-related disability. Based on established literature, MCID was defined as a reduction of ≥3.4 s in the TUG test and a reduction of ≥18 points on the DHI scale (28).

### 2.6 Statistical analysis

Continuous variables were presented as mean ± standard deviation (SD) or median with interquartile range (IQR), depending on the normality of the distribution assessed by the Shapiro–Wilk test. Categorical variables were expressed as frequencies and percentages. Missing longitudinal data (accounting for less than 8% of total data points) were handled using multiple imputation by chained equations (MICE), assuming data were missing at random. Because this was a retrospective consecutive cohort study, the sample size was determined by the number of eligible patients during the study period rather than by an *a priori* power calculation. A *post hoc* sensitivity analysis was performed for the multinomial regression component and is reported descriptively; this analysis was not used to validate the trajectory model structure. The adequacy of the GBTM solution was primarily assessed using BIC, AIC, average posterior probabilities, minimum group size, and clinical interpretability.

For baseline impairment phenotyping, candidate vestibular, gait, proprioceptive, balance-confidence, and functional mobility variables were first standardized as *z*-scores to place all measures on a comparable scale. Hierarchical clustering was performed using Ward's minimum-variance method with Euclidean distance. Candidate two-, three-, and four-cluster solutions were compared using cluster interpretability, group size, and internal validity indices, including average silhouette width. The final three-phenotype solution was selected before outcome modeling and was treated as an exploratory, data-driven exposure classification. Cluster labels were assigned according to the dominant impairment pattern within each cluster.

To identify heterogeneous pathways of functional recovery, we employed group-based trajectory modeling (GBTM), a specialized application of finite mixture modeling. The optimal number of trajectory groups was determined based on the Bayesian Information Criterion (BIC), the Akaike Information Criterion (AIC), average posterior probabilities of group membership (criterion >0.70), and clinical interpretability. For the primary trajectory analysis, models ranging from two to five groups were fitted using repeated TUG values with a censored normal distribution. A secondary, confirmatory trajectory analysis using DHI scores was performed to examine whether patient-reported vestibular disability showed a congruent pattern.

Once trajectory groups were established, multinomial logistic regression was utilized to determine the association between baseline impairment phenotypes (and other covariates such as age and CCI) and trajectory group membership, reporting odds ratios (ORs) and 95% CONFIDENCE INTERVALS (CIs). Furthermore, to define the exploratory “benefit thresholds,” we analyzed the dose-response relationship between cumulative FPRT hours and the probability of achieving the MCID as a planned secondary analysis. Because the relationship was anticipated to be non-linear, we applied Restricted Cubic Splines (RCS) with three knots located at the 10th, 50th, and 90th percentiles of the FPRT dose distribution, integrated within generalized estimating equations (GEE) to account for intra-subject correlation. In the RCS analysis, cumulative FPRT dose was treated as a time-varying exposure calculated at each assessment point. Therefore, thresholds below the nominal 50-h target reflect the cumulative exposure at which some patients had already achieved MCID during follow-up, rather than the final total dose prescribed for the 10-week program. Statistical significance was set at a two-tailed *P*-value < 0.05. All statistical analyses were performed using R software (version 4.2.2; R Foundation for Statistical Computing, Vienna, Austria) utilizing the lcmm, rms, and ggplot2 packages.

## 3 Results

### 3.1 Baseline characteristics of the study population

**Figure 1:**
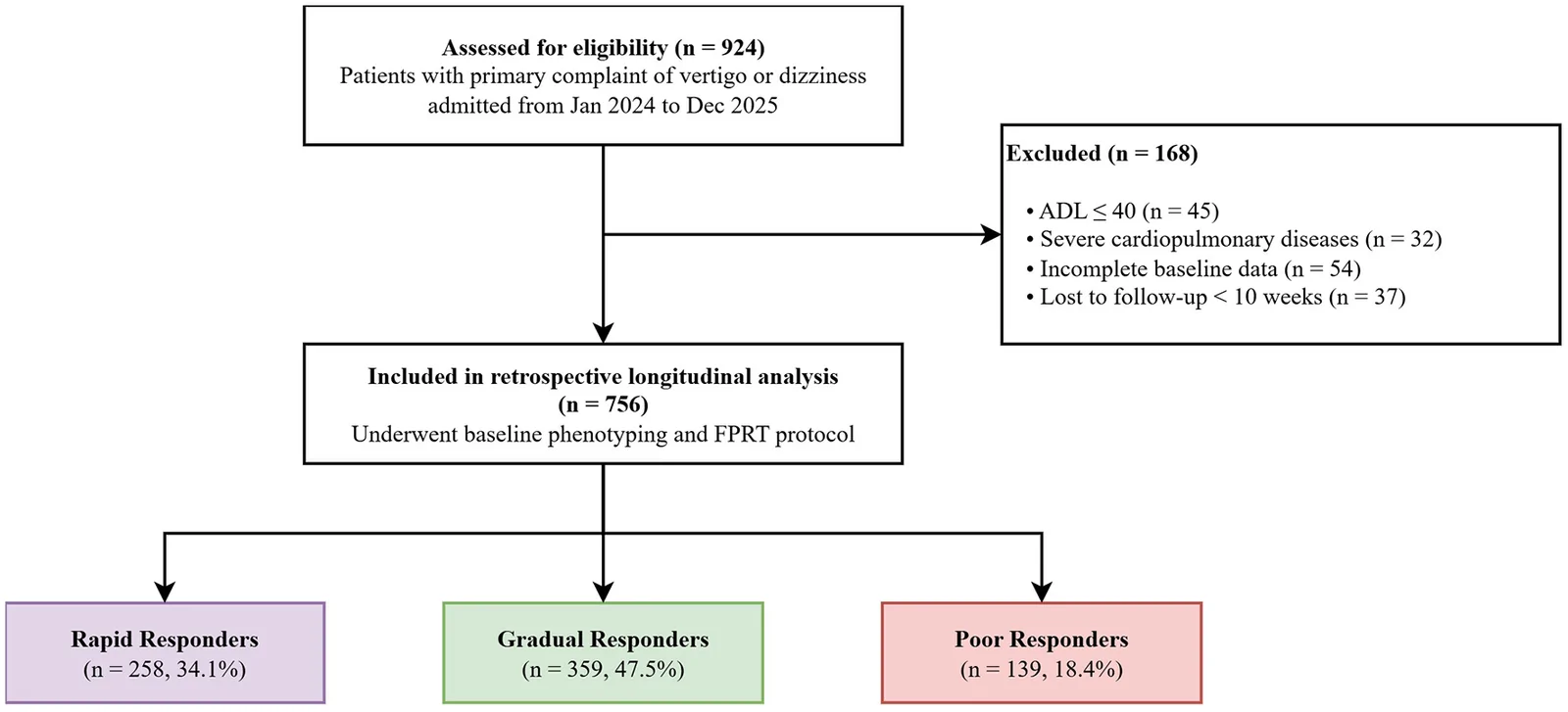
Patient selection flowchart. A STROBE-compliant flowchart detailing the screening, inclusion, and exclusion process of vertigo patients between January 2024 and December 2025. Out of 924 initially screened patients, 756 met the criteria for the final retrospective longitudinal analysis.

Between January 2024 and December 2025, a total of 924 consecutive patients with a primary complaint of vertigo or dizziness were screened. After applying the strict inclusion and exclusion criteria, 168 patients were excluded (45 had ADL ≤ 40, 32 had severe cardiopulmonary comorbidities, 54 had incomplete baseline data specifically missing vHIT results [*n* = 24], incomplete DHI questionnaires [*n* = 18], or unrecorded baseline TUG times [*n* = 12], and 37 were lost to follow-up before the 10-week mark). Consequently, a final cohort of 756 patients was included in the retrospective trajectory analysis. The patient selection process is detailed in the STROBE flowchart (Figure 1).

**Figure 2:**
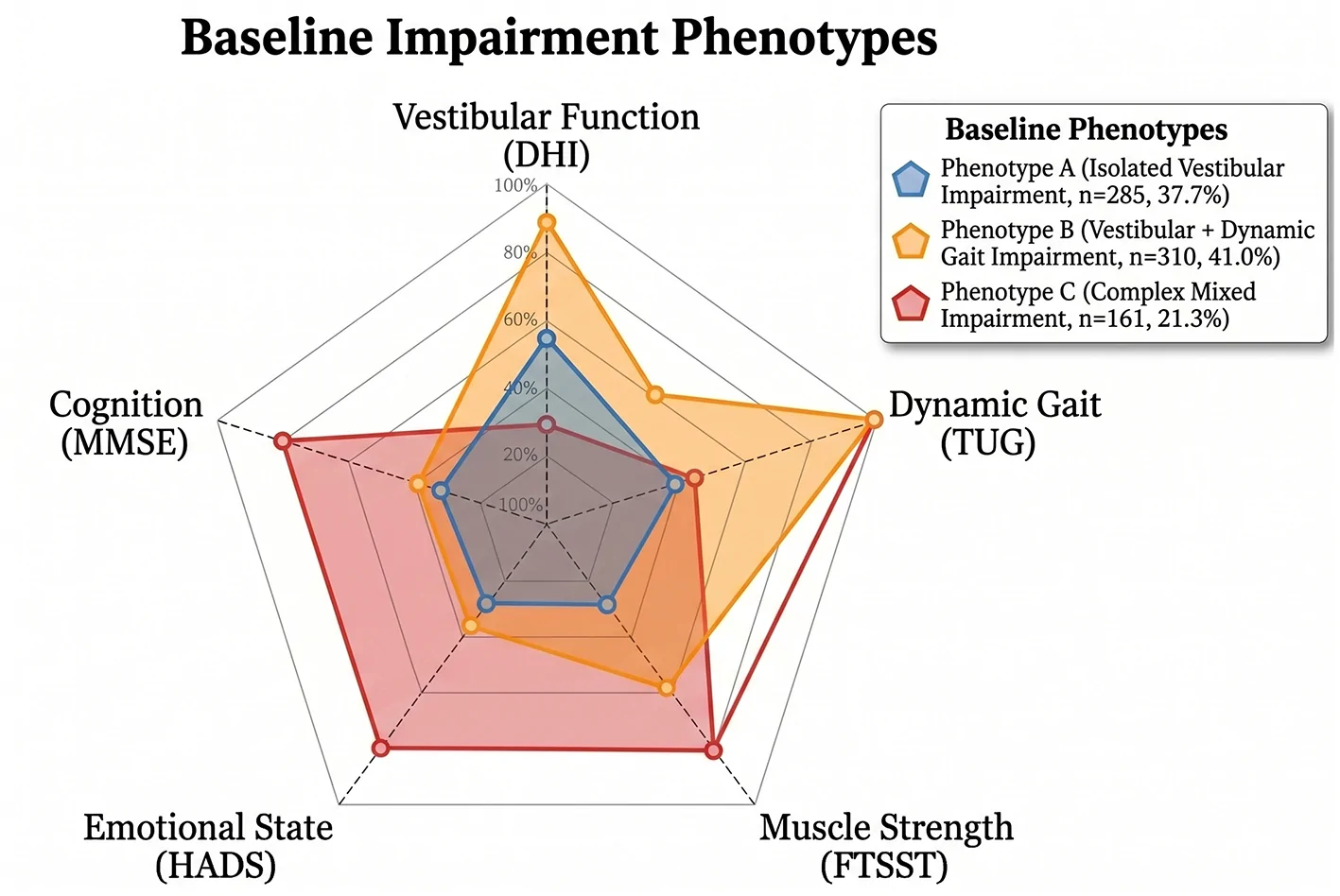
Baseline impairment phenotypes. A radar chart displaying the multidimensional baseline impairment patterns across the three identified phenotypes: phenotype A (isolated vestibular impairment), phenotype B (vestibular + dynamic gait impairment), and phenotype C (complex mixed impairment). Axes represent normalized scores for cognition, emotional state, vestibular function, dynamic gait, and muscle strength.

The mean age of the included cohort was 68.4 ± 8.2 years, with 408 (54.0%) being female. The median duration of vertigo symptoms prior to admission was 14 days (IQR 5–45 days). Regarding baseline functional status, the mean TUG test time was 15.6 ± 4.3 s, indicating a generally elevated risk of falls across the cohort. The mean DHI score was 58.4 ± 16.2, reflecting moderate to severe self-perceived handicap. Based on the comprehensive baseline assessment, hierarchical clustering identified three primary baseline impairment phenotypes: phenotype A (isolated vestibular impairment, *n* = 285, 37.7%), phenotype B (vestibular + dynamic gait impairment, *n* = 310, 41.0%), and phenotype C (complex mixed impairment involving vestibular, gait, and deep proprioceptive deficits, *n* = 161, 21.3%). The multidimensional baseline impairment patterns are visualized in the radar chart (Figure 2). Detailed baseline characteristics stratified by the subsequently identified trajectory groups are presented in Table 1. Baseline characteristics stratified by the baseline impairment phenotypes are additionally provided in Supplementary Table S1, because phenotype classification was used as the key exposure variable in the present analysis. Table 2 summarizes the distribution of specific neuro-otological diagnoses according to the Bárány Society Classification (38) across the three trajectory groups.

**Table 1:** Baseline characteristics of the study population stratified by trajectory groups.

Characteristics	Total (*N* = 756)	Rapid responders (*n* = 258)	Gradual responders (*n* = 359)	Poor responders (*n* = 139)	*P*-value
Age, years (mean ± SD)	68.4 ± 8.2	64.2 ± 6.5	68.8 ± 7.4	75.1 ± 6.9	< 0.001
Female sex, *n* (%)	408 (54.0%)	135 (52.3%)	198 (55.2%)	75 (54.0%)	0.782
Disease duration, days (median, IQR)	14 (5–45)	10 (4–30)	16 (6–50)	21 (10–65)	< 0.001
Charlson comorbidity index ≥3, *n* (%)	245 (32.4%)	52 (20.2%)	115 (32.0%)	78 (56.1%)	< 0.001
Baseline TUG, seconds (mean ± SD)	15.6 ± 4.3	12.8 ± 2.5	16.2 ± 3.4	19.4 ± 4.1	< 0.001
Baseline DHI score (mean ± SD)	58.4 ± 16.2	45.6 ± 12.1	61.5 ± 14.3	74.2 ± 11.8	< 0.001
Impairment phenotype, *n* (%)					< 0.001
Phenotype A (isolated vestibular)	285 (37.7%)	185 (71.7%)	85 (23.7%)	15 (10.8%)	
Phenotype B (vestibular + gait)	310 (41.0%)	60 (23.3%)	210 (58.5%)	40 (28.8%)	
Phenotype C (complex mixed)	161 (21.3%)	13 (5.0%)	64 (17.8%)	84 (60.4%)	

**Table 2:** Distribution of vestibular disorders according to Bárány Society Classification across Trajectory Groups.

Clinical diagnosis (Bárány Society)	Total (*N* = 756)	Rapid responders (*n* = 258)	Gradual responders (*n* = 359)	Poor responders (*n* = 139)
Benign paroxysmal positional vertigo (BPPV)	218 (28.8%)	134 (51.9%)	65 (18.1%)	19 (13.7%)
Vestibular neuritis/unilateral vestibulopathy	192 (25.4%)	85 (32.9%)	88 (24.5%)	19 (13.7%)
Vestibular migraine (VM)	114 (15.1%)	24 (9.3%)	78 (21.7%)	12 (8.6%)
Ménière's disease	68 (9.0%)	5 (1.9%)	46 (12.8%)	17 (12.2%)
Presbyvestibulopathy/bilateral vestibulopathy	96 (12.7%)	4 (1.6%)	35 (9.7%)	57 (41.0%)
Persistent postural-perceptual dizziness (PPPD)	68 (9.0%)	6 (2.3%)	47 (13.1%)	15 (10.8%)

### 3.2 Identification of distinct functional recovery trajectories

**Figure 3:**
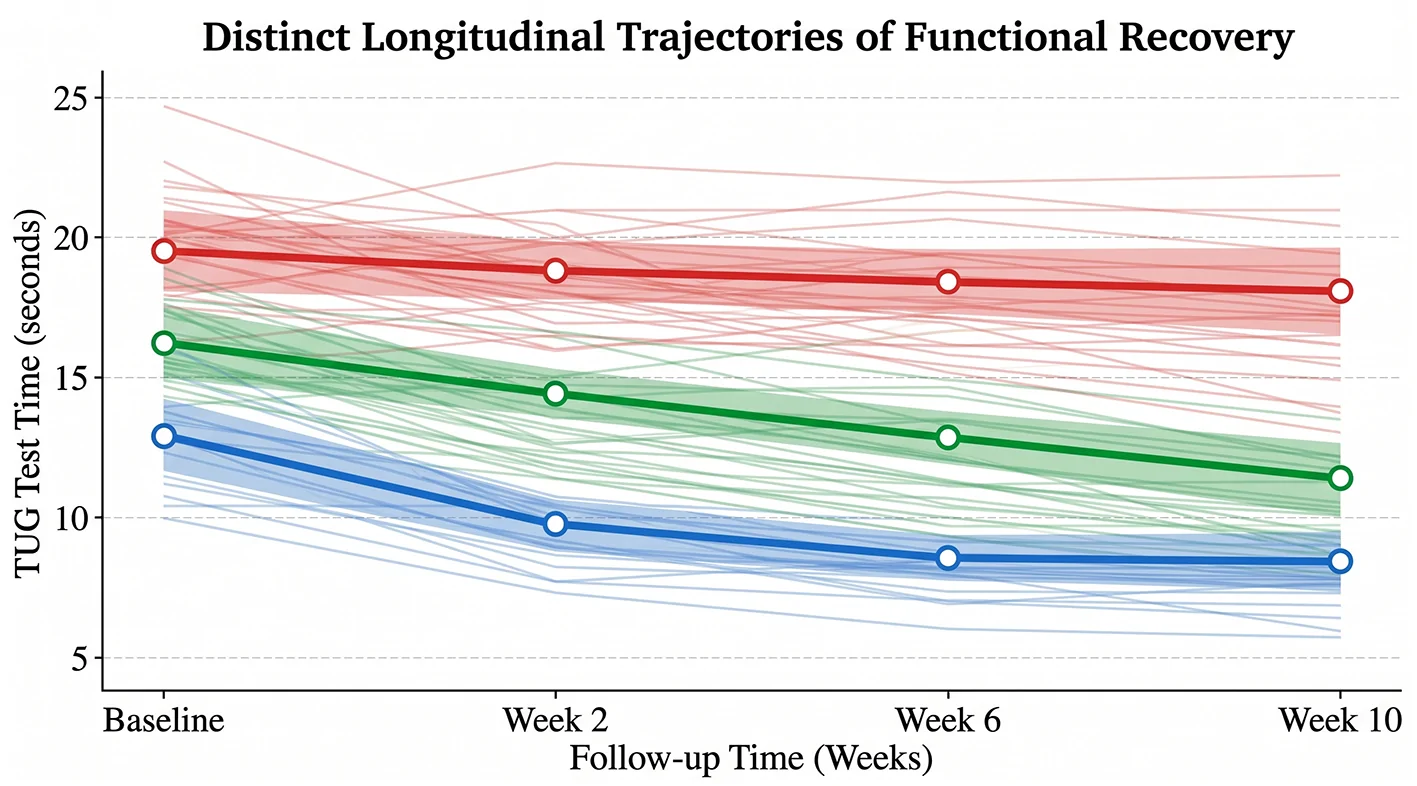
Distinct longitudinal trajectories of functional recovery. Spaghetti plot combined with group-based trajectory modeling (GBTM) showing three distinct recovery pathways over the 10-week FPRT period based on the timed up and go (TUG) test: rapid responders (blue), gradual responders (green), and poor responders (red). The shaded areas represent the 95% confidence intervals. *Lower TUG score indicates better functional mobility and lower fall risk.

Using GBTM based on the repeated measures of the TUG test over the 10-week period, a three-group model provided the optimal fit (lowest BIC and high posterior probabilities >0.85 for all groups). We identified three distinct longitudinal trajectories of functional recovery (Figure 3).

#### 3.2.1 Trajectory 1: “rapid responders” (*n* = 258, 34.1%)

Patients in this group started with relatively moderate baseline impairment (mean TUG 12.8 s) and exhibited a steep, early decline in TUG time within the first 4 weeks of FPRT, rapidly stabilizing near normal physiological limits (mean TUG 8.5 s at week 10).

#### 3.2.2 Trajectory 2: “gradual responders” (*n* = 359, 47.5%)

This was the largest group. These patients began with a higher baseline impairment (mean TUG 16.2 s). Their functional recovery curve demonstrated a steady, linear improvement throughout the entire 10-week period, eventually achieving a clinically significant reduction in TUG time by the end of the observation period (mean TUG 11.4 s at week 10).

#### 3.2.3 Trajectory 3: “poor responders” (*n* = 139, 18.4%)

This group was characterized by severe baseline impairment (mean TUG 19.4 s) and a remarkably flat recovery curve. Despite engaging in the FPRT protocol, these patients showed minimal reduction in TUG time, failing to reach the MCID threshold on average by week 10 (mean TUG 18.1 s). This subgroup predominantly consisted of older adults (mean age 75.1 years), heavily burdened with comorbidities (56.1% with CCI ≥3), and frequently exhibiting severe, complex mixed impairments (phenotype C).

Secondary analysis of DHI change showed a pattern broadly consistent with the TUG-based trajectories. Specifically, rapid responders' DHI scores fell steeply from a baseline of 45.6 to 15.2 at week 10; gradual responders improved from 61.5 to 28.4; whereas poor responders showed minimal meaningful change (74.2 to 65.8).

### 3.3 Determinants of trajectory group membership

Multinomial logistic regression was performed to identify the baseline determinants associated with trajectory membership, using the “rapid responders” as the reference category. The analysis showed that age, comorbidity burden, and baseline impairment phenotype were associated with trajectory membership. The complete multinomial logistic regression results are presented in Table 3.

**Table 3:** Multinomial logistic regression analysis of factors associated with trajectory membership (Ref: rapid responders).

Variables	Gradual responders OR (95% CI)	*P*-value	Poor responders OR (95% CI)	*P*-value
Age ≥ 75 years	1.85 (1.12–3.05)	0.016	3.45 (2.12–5.61)	< 0.001
Disease duration > 30 days	1.42 (0.95–2.12)	0.087	2.10 (1.35–3.28)	0.001
HADS Score > 11	1.65 (1.08–2.52)	0.021	2.35 (1.45–3.82)	< 0.001
15.9-7.8,-1.4498.8ptCCI ≥ 3	1.58 (1.05–2.38)	0.028	3.12 (1.85–5.25)	< 0.001
Phenotypes (Ref: phenotype A)				
Phenotype B	2.15 (1.45–3.18)	< 0.001	3.60 (1.95–6.65)	< 0.001
Phenotype C	3.85 (2.05–7.22)	< 0.001	4.85 (2.95–7.98)	< 0.001

**Figure 4:**
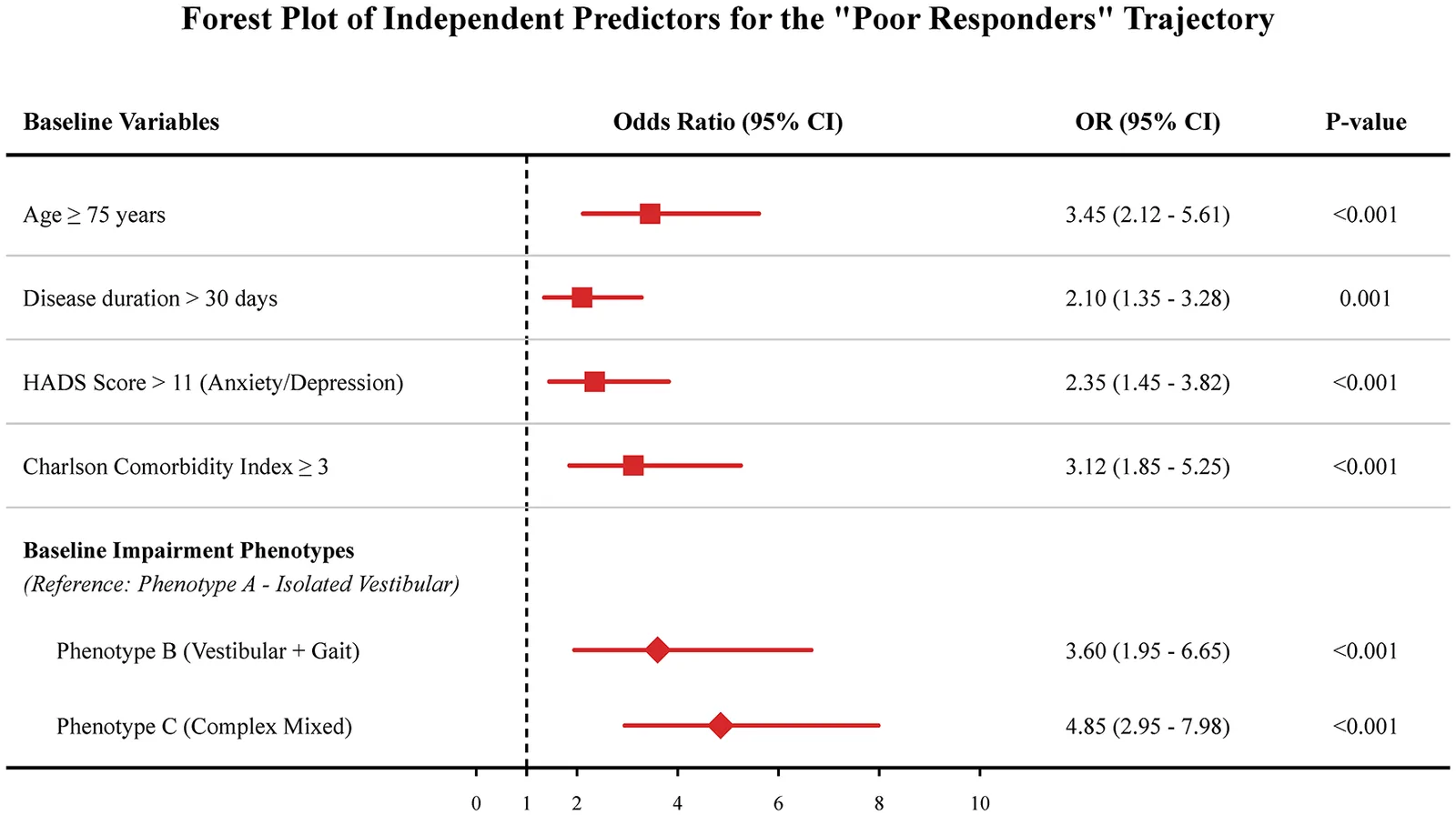
Forest plot of predictors for the poor responder trajectory. Multinomial logistic regression results showing variables associated with poor responder membership, with rapid responders as the reference. Advanced age and complex mixed impairment (phenotype C) were associated with higher odds of poor responder membership. Red squares/diamonds represent odds-ratio point estimates, and horizontal lines indicate 95% confidence intervals. An OR > 1 indicates an increased likelihood of membership in the poor responder trajectory.

Compared to patients with phenotype A (isolated vestibular impairment), those with phenotype B (vestibular + dynamic gait) had significantly higher odds of falling into the “gradual responders” trajectory (OR 2.15, 95% CI 1.45–3.18, *P* < 0.001). In addition, patients presenting with phenotype C (complex mixed impairment) were predominantly categorized as “poor responders” (OR 4.85, 95% CI 2.95–7.98, *P* < 0.001). Furthermore, advanced age (≥75 years) was associated with higher odds of Poor Responder membership (OR 3.45, 95% CI 2.12–5.61, *P* < 0.001). Baseline anxiety/depression (HADS score >11) was also associated with poorer trajectory membership, suggesting that psychosocial factors may be relevant to rehabilitation response (Figure 4, Forest Plot).

### 3.4 Non-linear dose-response relationships and benefit thresholds

**Figure 5:**
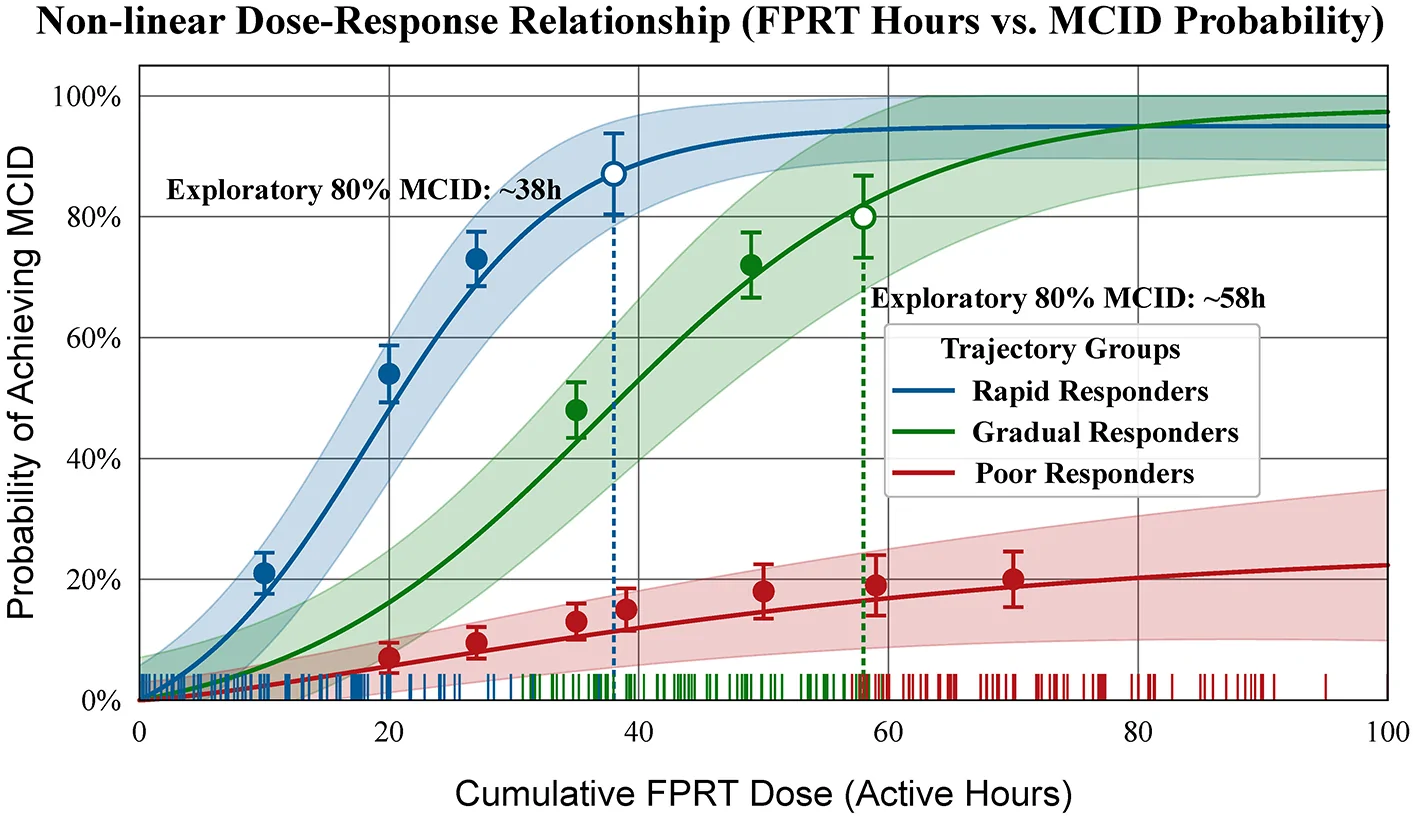
Non-linear dose-response relationship between FPRT hours and functional improvement. Restricted cubic spline (RCS) curves illustrating the varying probabilities of achieving the minimal clinically important difference (MCID) across different trajectory groups. The *x*-axis indicates cumulative active training hours, highlighting exploratory 80% MCID probability points for each subgroup. The lower rug plot shows the distribution of time-varying cumulative active training hours. Observed MCID achievement rates by dose interval are superimposed as points with binomial 95% confidence intervals, allowing comparison between empirical proportions and spline-estimated probabilities. Detailed raw data (*n*/*N*) for the observed MCID achievement rates across dose intervals are provided in Supplementary Table S2.

To explore potential dose–response patterns that may inform future rehabilitation planning, we analyzed the association between cumulative FPRT hours and the probability of achieving the TUG MCID (≥3.4 s reduction) using restricted cubic splines (RCS). The RCS curves demonstrated a clear non-linear relationship (P for non-linearity < 0.01) that differed across trajectory groups and baseline phenotypes (Figure 5).

These thresholds should be interpreted as exploratory time-varying dose points associated with MCID achievement during follow-up, not as recommendations to stop the full 10-week rehabilitation program. For the overall cohort, the probability of achieving MCID increased sharply as FPRT dose increased from 0 to 45 h, after which the curve began to flatten, indicating diminishing marginal returns. However, phenotype-stratified analysis revealed distinct thresholds: (1) rapid responders (predominantly phenotype A): the dose-response curve rose steeply early on. The inflection point (the threshold to achieve a >80% probability of MCID) was reached at approximately 35 to 40 cumulative hours. Doses beyond 45 h yielded negligible additional functional gains in this specific subgroup. (2) Gradual responders (predominantly phenotype B): the curve was less steep, necessitating a protracted period of training to accumulate benefits. The benefit threshold was significantly higher, requiring approximately 55 to 60 cumulative hours of FPRT to confidently cross the MCID boundary. (3) Poor responders (predominantly phenotype C, Elderly): the dose-response curve remained largely flat. Even with cumulative doses exceeding 70 h, the probability of achieving the MCID remained below 40%. This suggests that for patients with profound, multi-system deficits, standard FPRT doses are insufficient, and the threshold for benefit may exceed the conventional 10-week/50-h paradigm, or alternative interventions may be required.

## 4 Discussion

In this large-scale, retrospective longitudinal cohort study of 756 patients with vertigo, we aimed to delineate the functional recovery trajectories following fall prevention rehabilitation therapy (FPRT) and to quantify the specific dose-response relationships based on baseline impairment phenotypes. To our knowledge, this is one of the first studies to utilize group-based trajectory modeling (GBTM) combined with restricted cubic splines (RCS) to establish exploratory “benefit thresholds” for vestibular rehabilitation. We successfully identified three distinct functional recovery trajectories: “rapid responders” (34.1%), “gradual responders” (47.5%), and “poor responders” (18.4%). Furthermore, we demonstrated that the patient's baseline impairment pattern—ranging from isolated vestibular deficits to complex, multisystem dynamic gait impairments—was independently associated with their recovery trajectory and the estimated cumulative dose associated with clinically meaningful improvement (39, 40). Specifically, Rapid Responders reached an exploratory 80% MCID probability point at 35–40 h (often before the 50-h protocol completion), Gradual Responders required 55–60 h, whereas Poor Responders exhibited a plateau effect, highlighting the inadequacy of standard FPRT doses for severe phenotypes (41).

The observed heterogeneity in fall prevention rehabilitation therapy (FPRT) outcomes suggests that a uniform rehabilitation prescription may not adequately address the diverse impairment profiles observed in older patients with vertigo. The phenomenon of rapid recovery observed in our “rapid responders” group aligns with the principles of neuroplasticity and vestibular compensation (42). Patients with isolated vestibular impairment (Phenotype A) typically retain intact proprioceptive and visual systems. According to the sensory reweighting hypothesis (43, 44), these intact systems can rapidly up-regulate their contribution to postural control to compensate for vestibular deficits. Consequently, these patients reached their minimal clinically important difference (MCID) threshold relatively quickly, usually within 35–40 h of targeted therapy (45).

Conversely, the “gradual responders” (predominantly phenotype B, combining vestibular and dynamic gait impairments) and “poor responders” (phenotype C, complex mixed impairments with advanced age) require significantly more time and effort to adapt (46). Aging is intrinsically associated with progressive central nervous system degradation, including a decline in cerebellar processing efficiency and reduced somatosensory acuity, often linked to age-related white matter changes (47, 48). When a vestibular deficit is superimposed on pre-existing age-related white matter changes or peripheral neuropathy (as quantified by our mCTSIB assessments), the central nervous system's capacity for rapid sensory substitution is severely compromised. This may explain why phenotype C patients required substantially greater cumulative exposure yet often failed to achieve the MCID and plateaued prematurely. These findings are highly consistent with recent literature emphasizing the multifactorial etiology of falls in older adults and the necessity for prolonged, high-intensity interventions to overcome multisystem decline (49).

Our findings carry profound clinical implications for the precision management of vertigo and the operationalization of fall prevention strategies. First, the data suggest that relying solely on clinical symptoms is insufficient for prescribing vestibular rehabilitation therapy (VRT). A comprehensive baseline functional evaluation, incorporating objective metrics like the timed up and go (TUG) and dynamic gait index (DGI) which are validated measures for assessing fall risk and dynamic balance in vestibular disorders, may be useful for stratifying patients into distinct prognostic categories (50). By identifying a patient's functional phenotype early, clinicians can formulate realistic recovery timelines, thereby managing patient expectations and significantly improving adherence. For a patient with significant functional impairment, knowing upfront that meaningful improvement will likely require a substantial and sustained rehabilitation effort can prevent early drop-out due to perceived lack of progress.

Furthermore, these findings suggest that dedicated, multidisciplinary fall prevention and vestibular rehabilitation clinics may provide a useful framework for phenotype-informed assessment and follow-up. These specialized clinics can utilize defined functional thresholds to optimize resource allocation. Patients who are “Rapid Responders” could be fast-tracked to home-based, digitally monitored programs after a brief intensive clinical period, thus freeing up highly skilled therapists to manage the complex needs of “gradual” and “poor responders” through prolonged, supervised sessions. For the latter group, a generic, fixed-dose rehabilitation protocol is often inadequate. They may necessitate personalized adjunct therapies, such as cognitive-motor dual-task training, which has been developed to better assess and address the real-world deficits in individuals with vestibular dysfunction, to break through their rehabilitation plateau (51). Although direct evidence on dose thresholds in vestibular rehabilitation remains limited, broader rehabilitation-dose literature from other rehabilitation fields supports the general importance of dose, intensity, and frequency when interpreting treatment response.

Several limitations should be acknowledged. First, the retrospective, single-center design may introduce selection bias and limits the generalizability of the findings to different healthcare systems. Notably, the exclusion of 54 patients due to missing baseline variables might subtly alter response patterns. Furthermore, the vestibular diagnostic heterogeneity inherently limits broad assertions. Second, while we utilized daily video check-ins to monitor the dose (hours) of home-based FPRT, the exact intensity and kinematic quality of the exercises performed at home remain difficult to quantify perfectly, potentially introducing unmeasured confounding, rendering cumulative hours a proxy rather than an absolute measure of rehabilitation intensity. Third, the observational nature of the study precludes absolute causal inferences. Fourth, the baseline impairment phenotypes derived in this study require rigorous external validation before widespread clinical adoption. Future large-scale, multicenter, randomized controlled trials stratifying patients by baseline phenotype are required to prospectively validate these benefit thresholds.

## 5 Conclusion

Baseline vestibular, dynamic gait, and proprioceptive impairment phenotypes were independently associated with longitudinal recovery trajectories and exploratory dose–response patterns in patients with vertigo undergoing FPRT. These findings suggest that phenotype-informed rehabilitation planning may help refine clinical expectations and generate hypotheses for individualized dosing strategies. The exploratory thresholds observed in this retrospective cohort, approximately 35–40 h for milder deficits and 55–60 h for gradual responders, should be considered hypothesis-generating and require prospective validation before being used as definitive prescription targets.

## Data Availability

The original contributions presented in the study are included in the article/Supplementary material, further inquiries can be directed to the corresponding authors.
