# Supplementary material for: Dose–response and recovery trajectories of fall prevention rehabilitation in older patients with vertigo: a retrospective cohort study based on vestibular and gait impairment phenotypes
Source: Front Neurol. 2026 Aug 17;17:1874677. doi: 10.3389/fneur.2026.1874677 (PMC13526622; doi:10.3389/fneur.2026.1874677)
Supplement: Supplementary file 1 [file Data_Sheet_1.docx]

#### Supplementary Table S1. Baseline Characteristics Stratified by Baseline Impairment Phenotypes

| **Characteristics** | **Phenotype A (n=285)** | **Phenotype B (n=310)** | **Phenotype C (n=161)** | **P-value** |
| --- | --- | --- | --- | --- |
| Age, years (mean ± SD) | 65.1 ± 6.8 | 69.2 ± 7.1 | 74.6 ± 7.5 | <0.001 |
| Female sex, n (%) | 154 (54.0%) | 168 (54.2%) | 86 (53.4%) | 0.978 |
| Charlson Comorbidity Index ≥3, n (%) | 61 (21.4%) | 101 (32.6%) | 83 (51.6%) | <0.001 |
| Baseline TUG, seconds (mean ± SD) | 13.1 ± 2.8 | 16.5 ± 3.6 | 19.8 ± 4.4 | <0.001 |
| Baseline DHI score (mean ± SD) | 47.2 ± 13.5 | 62.8 ± 14.8 | 75.4 ± 12.6 | <0.001 |
| mCTSIB Condition 4 Fall Rate, n (%) | 28 (9.8%) | 145 (46.8%) | 124 (77.0%) | <0.001 |
| Dynamic Gait Index (DGI) score (mean ± SD) | 20.4 ± 2.1 | 15.8 ± 2.5 | 12.1 ± 2.8 | <0.001 |
| Functional Reach (FR), cm (mean ± SD) | 31.2 ± 4.5 | 25.4 ± 5.1 | 19.8 ± 4.8 | <0.001 |
| Five Times Sit to Stand Test (FTSST), sec (mean ± SD) | 11.2 ± 2.4 | 14.5 ± 3.1 | 18.2 ± 4.2 | <0.001 |
| Single Leg Stance (SLS) time, sec (mean ± SD) | 18.5 ± 6.2 | 11.4 ± 5.8 | 5.2 ± 3.1 | <0.001 |
| Activities-specific Balance Confidence (ABC) score (mean ± SD) | 68.5 ± 12.4 | 52.1 ± 14.2 | 35.4 ± 11.8 | <0.001 |
| Fall Risk Questionnaire (FRQ) score ≥4, n (%) | 114 (40.0%) | 217 (70.0%) | 148 (91.9%) | <0.001 |
| Abnormal vHIT (gain <0.8 or corrective saccades), n (%) | 242 (84.9%) | 223 (71.9%) | 132 (82.0%) | 0.001 |
| Caloric weakness (>25% asymmetry), n (%) | 198 (69.5%) | 170 (54.8%) | 105 (65.2%) | <0.001 |
| Dynamic Visual Acuity (DVA) abnormality (loss >2 lines), n (%) | 156 (54.7%) | 232 (74.8%) | 142 (88.2%) | <0.001 |
| Joint Position Sense abnormality (lower limb), n (%) | 15 (5.3%) | 85 (27.4%) | 112 (69.6%) | <0.001 |

#### *Note: P-values were calculated using one-way ANOVA or Kruskal–Wallis tests for continuous variables and χ² tests for categorical variables, as appropriate.*

#### Supplementary Table S2. Observed MCID Achievement Rates by Cumulative FPRT Dose Intervals and Trajectory Groups

| **Dose Interval (Active Hours)** | **Rapid Responders (n/N, %)** | **Gradual Responders (n/N, %)** | **Poor Responders (n/N, %)** |
| --- | --- | --- | --- |
| 0 - 20 | 25 / 118 (21.2%) | 6 / 85 (7.1%) | 3 / 48 (6.3%) |
| 21 - 40 | 168 / 230 (73.0%) | 68 / 142 (47.9%) | 8 / 62 (12.9%) |
| 41 - 60 | 55 / 62 (88.7%) | 185 / 258 (71.7%) | 7 / 38 (18.4%) |
| 61 - 80 | - | 152 / 164 (92.7%) | 5 / 26 (19.2%) |
| 81 - 100 | - | 36 / 37 (97.3%) | 2 / 10 (20.0%) |

#### *Note: Values represent patient-assessment observations rather than unique patients; therefore, a single patient could contribute observations to more than one cumulative-dose interval. n/N represents the number of observations achieving MCID over the total number of observations in that dose interval.*
